# Strategic directions in the *what* and* how* of learning and teaching innovation—a fifty-year synopsis

**DOI:** 10.1007/s10734-022-00945-2

**Published:** 2022-10-20

**Authors:** R. A. Ellis

**Affiliations:** Office of the Pro Vice Chancellor, Griffith University, Nathan campus, Bray Centre (N54) Room 2.24, Nathan, QLD 4111 Australia

**Keywords:** Learning, Teaching, Innovation, Outcomes, Process, Online

## Abstract

Student learning experiences at university are constantly evolving; new disciplinary discoveries, new knowledge, interdisciplinary synergies and new exigencies make learning a dynamic experience for students, teachers and researchers alike; and that is just the *what* of learning. Add to this, changes in the *how* of learning, new pedagogies and new technologies, new partners in the provision of learning, as well as new configurations of where learning takes place, such as on campus, at home, in the workplace and online; and it is not hard to make the case that learning experiences of students enrolled in a degree are relatively more complex today than they were even 20 years ago. Much of this change has been captured over the last five decades in the journal *Higher Education.* The ongoing challenge of these changes is the complexity that accompanies them. How do we improve the student experience of learning in a complex context? What should the outcomes of a higher education degree be? What learning processes are likely to lead better outcomes? How do you assess the quality of learning that may occur in small groups on campus or online, or in large groups in both places, or in laboratories or the workplace? What is the role of material objects in these experiences and do they contribute to outcomes? This manuscript will consider such questions and where the journal is pointing researchers towards new avenues that are developing in learning and teaching internationally.

## Introduction

The journal of *Higher Education* has captured key issues and concepts over the last 50 years that help to explain current themes and debates in the international higher education sector. Without playing a role to facilitate these discussions in a scholarly way, the quality and progress of the sector today would be impoverished. To describe how the journal of *Higher Education* has played this role, this paper groups significant contributions to the international debate from the journal’s publications into the outcomes of the student experience of learning, or the ‘what’, and material aspects of the student experience, or part of the ‘how’ of learning and teaching. The last section of the paper looks at journal articles investigating associations between the how and what of learning and teaching.

*Higher Education* has always sought to attract and publish diverse views on the values, design, implementation and innovation of post-secondary education. This is evident from reading the cited articles in the reference list at the end of this paper. Together they suggest that the ongoing innovation through experimentation in learning and teaching is a key part of the sector. It is motivated by both theory and practice and the context in which it occurs. It typically involves dimensions of quality, sustainability, and increasingly, material aspects like technology and is most productively evaluated in relation to performance and outcomes. Even a brief review of the articles referenced in this paper reveals that it is increasingly important and difficult to analyse and understand as the complexity and interdependence of variables involved in learning and teaching grows.

### Defining innovation in the context of the journal

In the context of the journal’s articles, innovation can mean slightly different things depending on the topic. The following describes different ways articles in the journal contextualize the idea of innovation.

In an early study (Eraut, 1975), the authors put forward innovation as the challenges in establishing an interdisciplinary agenda for universities in the context of traditional organizational structures which tended to separate expertise rather than look for synergies. In the 1970s, a manuscript suggested a government-led conference in Poland setting out its agenda for universities to research the quality of learning and teaching for a decade as an example of innovation in the sector (Kupisiewicz & Januskiewicz, 1974). In the 1980s, a Brazilian study reported on innovative reforms of a Presidential Commission on Higher Education, emphasizing the value in different types of HE institutions and purposes, and the value of encouraging their autonomous operations (Schwartzman, 1988). In the 1990s, with the burgeoning middle class, India set about a number of reforms and innovations in their HE sector in order to meet growing demand for education (Altbach, 1993).

Since the 2000s, a key theme of innovation in learning and teaching has involved the inclusion of material elements in the student experience of learning. In part, these have grown with the introduction of personal computing world-wide since the 1990s and taken holistically, can offer a way into innovation in learning processes. Materiality in learning and teaching can be understood as *the negotiated processes through which the material becomes entangled with the social to bring forth actions, subjectivities and ideas* (Fenwick & Edwards, 2014, p.1). While the growth has been transparent in the sense that we can track its increasing frequency of use through numerous journal publications, university annual reports, government investigations and the like over the last twenty years, particularly in the last couple of years in the pandemic (Flores et al., 2022; Komljenovic, 2020), understanding the extent of its contribution to knowledge creation and learning outcomes remains a relatively under-investigated area, and an important avenue for future research.

Looking at the concept of ‘innovation’ across the decades in the journal demonstrates key values and strengths of the journal. The choice of papers published acknowledges the diversity across the sector, rather than attempting to impose homogeneity. The published articles recognize that while experiences of learning and teaching are culturally embedded, there are some common elements which transcend cultural boundaries and can be used to understand what might be otherwise impenetrable problems. Furthermore, the choice of papers shows that the journal content is not Euro-American centric, but rather welcomes papers from all continents. This is one of the strengths of the journal and its place in the international debates.

While there are different emphases on innovation in the learning and teaching agenda in the journal, the common idea drawn on in this manuscript is innovation as an ongoing part of experimentation to improve learning and teaching, in contrast to innovation as more of an abrupt change and disruption to a field (Serdyukov, 2017).

## Outcomes arising from the university student experience—part of the *what* of learning

Much innovation in the learning experience of university students has revolved around transferable, measurable outcomes of a university education. This has been in part driven by government policy and also by a concern for minimum thresholds in the quality of a university degree (Darling et. al., 1989; Dill et al., 2013; Geva-May, 2001). It has taken many forms including how graduate attributes are related to generic skills, work-integrated learning and global citizenship skills.

### Generic skills

Part of the innovation lens passed over universities includes the types of skills with which graduates are expected to enter industry. A continuing debate about university students has been whether graduates can measurably display generic attributes. This debate considers the extent to which attributes such as teamwork for problem-solving can be regarded and measured as a transferable attribute of a graduating university student, even if the graduates come from diverse disciplines.

Internationally, a challenge across higher education systems in most continents has been the extent to which a comparable framework can be developed. A precursor to generic skill measurement is the development of models related to learning outcomes that have validity and reliability in the eyes of practitioners (Bennett et al., 1999). For example, ethical frameworks can be used to bind interpersonal and citizen-like attributes of university graduates across multiple higher education systems into a framework that offered the possibility of more commensurate measurements (Boni & Lozano, 2007).

Some studies looked at indirect relationships amongst graduate attributes and the characteristics of the physical and disciplinary learning environments of those students (Vaatstra & De Vries, 2007). The results were reported in three areas; graduates from active learning environments tended to display generic and reflective attributes, particularly those from disciplinary majors that emphasize active design in their curricula. The features of the activities that seemed to most engender these reflective outcomes in graduates included a willingness to work in teams as well as alone and strong analytic skills. However, no matter the discipline, it seems that the development of effective generic skills is inextricably entwined within a learning environment shaped by active learning and the practice of those skills in meaningful engagement (Kember, 2009).

### Work-integrated learning (WIL)

A particular developmental context for generic skills has been the integration of experiences of work-integrated learning for university students. Most governments encourage close cooperation between universities and industry and many have put in place financial and accreditation incentives to strengthen the relationships. This is in part driven by the concept of universities being part of the engine house of economies and is in some considerable tension between universities as engines of economies versus all-round student development as persons, as both are about learning and teaching. However, research into the challenges of integrating experiences of work-integrated learning in the programs of university students has revealed it is not always an easy fit (Pinto & Pereira, 2019).

Some of the challenges arise if students are not ready for the demands of a workplace. Balancing being directed and showing initiative is not an easy skill to develop in an unfamiliar context. This skill along with the relevance between the WIL experience and the student’s study, the willingness to commit to the placement, and the outcomes focus of the student are other challenges which need to be addressed (Valo, 2000).

These challenges can be exacerbated in part by the underlying concept of work-integrated learning. If it is conceived of as an afterthought, something bolted onto a curriculum, then it often does not make much sense to students who also perceive it to be unrelated to their program goals (Björck, 2021). In contrast, if it is designed into the curriculum, with activities which help to *transition in* students to thinking about their professional identity at the beginning of their degree, with *transition through* activities which help them to explore how their skill development will enable a professional career, and *transition out* activities which prepare students for a world of practical work, then research has shown it is much more meaningful for students and their academic experience and performance (Jackson, 2017; Jackson & Collings, 2018).

For international students, the challenges in WIL experiences are often more complex than for domestic students. In addition to getting placements at all, the challenges of speaking and writing in English in ways appropriate for the workplace in which they are working, and adapting to the culture of the workplace, can be a sufficiently serious impediment to make a placement unworkable (Lee et. al., 2021). The challenges for international student experience also extend to supervisors. They can often find it difficult to assess the competence of international students, particularly designing assessment schedules that work for both the students and the employers offering the placement (Jackson & Bridgstock, 2021).

### Global citizenship

A particular graduate attribute emphasized by many universities is global citizenship skills, which can be defined as:

International awareness and openness to the world, based on appreciation of social and cultural diversity, respect for human rights, and dignity and Understanding and appreciation of social, economic, or environmental sustainability issues and Leadership capacity, including a willingness to engage in constructive public discourse, and to accept social and civic responsibility. (Kirk et. al, 2018, p.4)

Initially, global citizenship skills were conceptualized more from the point of view of international students and their integration into their higher education experiences in their visiting countries and the economic benefits that ensued (Spencer-Oatey & Dauber, 2019). However, a number of studies have noted the cultural benefits that accrue from an institutional population in universities in terms of intercultural learnings and diversity for visiting students (Singh & Jack, 2018) and for both students and teachers (Huang & Horiuchi, 2020). For example, a government-led initiative in Korea has been the introduction of foreign professors into the classroom in an attempt to improve the internationalization of the curriculum (Jang, 2017).

It is in the context of the above ideas that institutions initially pursued curriculum internationalization as a means to provide support for international students and ensure their success through induction into western higher educational methods and practices (Clifford 2009, 2011; Sawir 2011, 2013). More recently, institutional understandings of internationalizing the curriculum have shifted—at least in the terms by which strategies are articulated—to include focus on internationalization for the whole student body (Clifford 2009; Sawir 2011, 2013). This has been associated with a drive to increase outbound mobility (Clifford, 2011), and to ensure opportunities are available for students to develop globally relevant skills. These broader conceptions arise from the belief that universities are obligated to make all of their students aware of their role as global citizens in an increasingly internationalized, multicultural world and to prepare them for success in the global labour market (Clifford 2009, 2011).

The above paragraphs offer a brief synopsis of some of the key outcomes or *what* of learning and teaching over the last few decades. There is no doubt that debate will continue about the most appropriate outcomes that we should be seeking from a university education. Significantly, *how* a university education is provided has been changing remarkably over the last twenty years, largely enabled by *material* affordances, and motivated by new ways of discovering and disseminating knowledge and skills acquisition.

## Material aspects in the university student experience—part of the *how* of learning

In the context of this synopsis, the *how* of learning focuses on those material elements which contribute to the process of learning and how students, teachers and educational leaders approach them, to contrast with the elements described in the first part of this manuscript which can be conceived of as the outcomes or *what* of learning. A further distinction between the two in the context of the journal’s history is that in a high level of analysis, there is relatively more consensus across the field on the structure of outcomes from the university experience of learning than on the structure of processes in the experience of learning, for example, how technology is best described in the processes of learning and in a university organizational structure. The following review of research on the role of material elements in the processes of learning helps to clarify these tendencies.

A noticeable focus of research in the journal has been the role and potential contribution of material aspects in the process of learning such as technology and its cross-disciplinary role as a part of the fabric of a university, as opposed to the role of technology delimited by disciplinary boundaries. A particularly insightful comment was made in the early 1970s by a researcher considering innovation in education and interdisciplinary approaches.

How far has the university gone in penetrating the education/innovation system? Clearly not very far yet. In particular, the education function of the university was not capable of adjusting to the requirements of inter-disciplinary organization beyond the level of elementary technology. To a large extent, education in technology is still categorized by disciplines and departments called “mechanical engineering,” “electrical engineering,” "chemistry," etc. This has led to two grave consequences: one is a schism between the education and research functions of the university at levels of higher interdisciplinary organization, which is already becoming a problem at the level of complex technical systems; university research and development in these areas is increasingly set up and carried out outside the educational structures. The other consequence is a growing mismatch between…education and the requirements of industry which is reorganizing itself in terms of technological or even sociotechnological systems tasks…no wonder, then, that the particularly strong systemic interaction between man and computer has not yet found a place in the university. (Jantsch, 1972, p. 22)

Using the journal’s publications as a barometer of international attention on technology as an increasingly ubiquitous part of the process of learning across *all* disciplines, it would be more than twenty years later before the affordances and availability of fit for purpose personal technologies started to appear in ways that would sustain a relatively ‘stronger systemic interaction between (student) and computer’ in the university as an organizational concept (Beattie & James, 1997; O'Shea, 2004). This observation does not mean that in the interim, a growth of educational technology within particular disciplines or attempts at organizing university-wide educational technology support for did not occur. Rather, the developments between the 1970s and 2000s were essential to the ubiquitous integration of technology as an enabler of university strategy that has occurred in the last couple of decades (Guo & Huang, 2020).

In the 1970s and 1980s, universities often emphasized audio, audio-visual and television networks in student support services. Such services were coordinated at the level of a university (Austwick & Harris, 1972) for the purposes of coping with quality and quantity of educational service provision (Correa, 1972; Tow & Phillips, 1982). In the 1990s, the development of personal computers saw growth of ‘flexible’ learning and teaching as an approach to design of the student experience and course delivery (Anderson & Garrison, 1995; Beattie & James, 1997; Mowrer, 1996). With the benefit of hindsight, all of these developments can be seen leading to the growth of online learning in the university student experience, now probably understood as a constituent part of the experience which is unlikely to disappear given the history to date and the recent role it has played in the pandemic (Flores et al, 2022).

### Online learning in the university student experience

Online learning is emphasized in this synopsis because of the significant structural change it brings about to the university student experience of learning. When used productively in blended course designs, it typically divides activities across separate learning environments (face-to-face and online), which can create difficulties for alignment with learning outcomes and student understanding. This structural change to the learning environment (that is that part of the experience can be put into a separate virtual place with its own affordances different to a classroom) suggests that particular approaches to course design, teaching and evaluation are required to both account for the difficulties of fragmented learning space and, at the same time, benefit effectively from the affordances of an integrated learning environment that cannot be achieved in a face-to-face setting alone (Flores et. al., 2022; Lohmann, et. al., 2019).

‘Online learning’ as a conceptual part of the university student experience was not used in a manuscript title in the journal until the mid-2000s (Wen & Tsai, 2006), although it first appeared in the text of a manuscript in the 1990s (Hardy et al., 1994). Since then, key issues that have been debated include the following: the pedagogy of online learning and teaching (Gonzalez, 2009; Goodyear et. al., 2005; McConnell, 2018; Park & Choi, 2014; Snowball, 2014), including gaming as a way of learning and teaching (Cheville, 2016; Coleman & Money, 2020); the importance of students’ attitude and motivation to online learning (Cho et al., 2021; Ferrer, et. al., 2020; Nordmann et. al., 2022); how to leverage the digital affordance of the online environment to maximize the benefits of student feedback on learning (Espasa & Meneses, 2010; Hellings & Haelermans, 2020; Ibabe & Jauregizar, 2010); assessing students in online and blended learning environments (Carless, 2015; Demir, 2018; Hernández, 2012), including peer learning (De Backer et al., 2015; De Smet et. al., 2010; Wen & Tsai, 2006); and teaching management issues such as dealing with the massification of higher education and the impact on equity and quality (Hornsby & Osman, 2014; Maringe & Sing, 2014), and policy issues arising from a use of online learning in the university student experience (Barratt-Pugh et. al., 2019; Han, 2020; Komljenovic, 2020; McConnell, 2018). While there is insufficient room in this manuscript to deal with these issues individually, together they demonstrate the growing fundamental role the online experience is playing in the university student experience of learning, particularly magnified during the recent pandemic (Flores et. al., 2022; Guppy et. al., 2022).

Associations between the *how* and *what* of learning and good teaching have long been part of the international research agenda into learning and teaching (Biggs, 1979, 1996; Entwistle & Entwistle, 1991; Kember, 2000; Prosser & Trigwell, 2014; Ramsden, 1983). With the advent of materiality becoming a foundational part of the experience, interest in the relationships between these key aspects of the student experience and the quality of learning outcomes continues to be a key focus of debate and research.

## Associations between the *what* and *how* of university student learning

To discuss the significance of the ways in which the *what* of learning seems to be related to the *how*, the following adopts a student perspective on the phenomenon. The adoption of this perspective facilitates a relational and student-centred understanding of learning and teaching, as the ways students report thinking about, doing, perceiving and feeling about their experience of learning can be measurably related to relatively deeper ways that some teachers approach their course design and teaching (Prosser & Trigwell, 2014). This insight is magnified in value when one understands how student conceptions of what they are learning and how they perceive the environment in which they are studying (Entwistle, 1991; Ramsden, 1979) offer a way into the quality of how they approach their learning (Biggs, 1979, 1996) and the quality of their outcomes (Trigwell et al., 1999; Guo et. al., 2022). In other words, if we know how to improve the processes of learning, we are more likely to improve the products or outcomes of learning.

Key to understanding the outcomes of learning discussed in the first part of the manuscript are the underlying conceptions of learning held by students. Student conceptions of learning have held an important part in the research agenda into university learning experiences as the qualitative differences of conceptions reported by students have been found to be related to how they approach their learning with and without technology, the overall quality of their experience and academic performance (Eklund-Myrskog, 1998; Flores et. al., 2022; Makoe et al., 2008; Trigwell & Prosser, 1991).

One of the earliest studies reported in the journal reviewed the seminal work undertaken by Marton and others in the Gothenburg group (Gibbs et al., 1982). This review put forward an evidence-based case and accompanying theoretical framework of how student conceptions of learning were related to approaches to learning and qualitatively different learning outcomes. Broadly summarizing, cohesive conceptions of learning, those that report ideas about learning which are most closely aligned to the learning outcomes of the course and the development of understanding, tend to be related to deep approaches to learning, those approaches which engage in activities in order to seek the underlying meaning through methods such as critical analysis, reflection, cross-referencing and synthesis of key ideas. In contrast, fragmented conceptions of learning, those that reveal little if any awareness of understanding of the subject matter and learning outcomes of a course, tend to be related to surface approaches to learning, which adopt relatively more formulaic and mechanistic strategies. Subsequently, these associations were found to be consistent with, and logically related to, the student experience of learning in blended contexts in which part of the experience is online (Ellis et al., 2012). Since the 1970s, there have been numerous studies which have pursued these associations in student learning research with remarkable coherence in terms of theoretical development, face validity and statistical significance (Cano et. al., 2018; Cervin-Ellqvist et. al., 2021; Eklund-Myrskog, 1998; Entwistle, 1991; Entwistle, 1997; Hounsell, 1979; Heikkilä, 2011; Herrmann, 2014; Kember, 2000; Makoe et al., 2008; Marton and Svensson, 1979; Mathias, 1980;; Norton & Crowley, 1995; Postareff, 2017; Richardson et al., 1999; Richardson, Trigwell & Prosser, 1991; Tait and Entwistle, 1996; Trigwell et al., 1999; Van Rossum et al., 1985; Wierstra et. al., 2003).

### The measurement of learning experiences

The journal *Higher Education* provides a seminal evidence-base of approaches to measuring the quality of the learning experience and outcomes in higher education. An illuminative way to enter research outcomes of the measurement of the learning experience and its outcomes is to look at the instruments reported on in the journal. Some of the earliest research in the journal reported on subscales investigating the student experience of learning by Entwistle and Wilson, Marton and Pask and Biggs (Entwistle et al., 1979). A key dimension of that debate recognized that student approaches to learning was a key aspect which needed to be understood, but the parts of that subscale captured in individual items on different research questionnaires varied from researcher to researcher. Some of this variation involved how the subscales measured orientation to learning, whether the approach (strategies and intention) was deep, surface or achieving, whether processes of learning tended towards comprehension or memorization and to what level of quality outcomes these variations were most likely to lead. This debate led to the development of a number of distinctive approaches to measurement in the field.

A well-known approach to measuring the student experience revolves around the idea of structured learning outcomes (Biggs, 1979), which would later form part of a foundational approach to quality in learning and teaching referred to as constructive alignment (Biggs, 1996) and the surveys which under pinned the idea such as the Study Process Questionnaire (Gow & Kember, 1990). Biggs and others put forward a structure that helped many ensuing research approaches to make a qualitative distinction between different outcomes of the learning experience (for example, Housell, 1979; Richardson et al., 1999; Trigwell and Prosser, 2014). In this context, surveys and questionnaires continued to be developed amongst a broader debate on issues such as whether qualitatively different outcomes were more closely related to cognitive styles, or the subject matter being studied and the assessment activities employed (Thomas & Bain, 1982).

Another approach to measuring the student experience of learning was to look at student perceptions of the learning environment. Drawing on the work of Entwistle, Biggs and others, Ramsden looked at perceptions of learning in a research approach to the student experience which eventually led to the development of the Course Experience Questionnaire [CEQ] (Lawless & Richardson, 2002; Rasmden, 1979; Trigwell & Prosser, 1991). The CEQ was used by every Australian University for more than a decade starting from 1993 and influenced national approaches to survey development in the UK (Sun & Richardson, 2012). The idea behind the perceptions’ subscale of Ramsden was that positive perceptions of key aspects of the environment like student perceptions of the quality of teaching, appropriateness of assessment and workload and the like were likely to be significantly related to qualitatively higher levels of academic performance. This proved to be the case in the literature cited here and elsewhere and perceptions of learning have been confirmed as a constituent part of the research agenda into university student learning since then.

There are many other instruments helpful in understanding the quality of the student experience of learning. The ‘Approaches to Study Inventory’ of Tait and Entwistle does similar work to that of Biggs and others with slightly different purposes in different versions (Entwistle & Tait, 1990). For example, one version was redesigned to highlight students who reported a relatively weak approach to learning (Tait & Entwistle, 1996). Another drawing on the work of Biggs, Prosser and Trigwell looks at the role of materiality and how it can be effectively located and evaluated in the student experience of learning (Ellis, Bliuc & Goodyear, 2012; Fenwick & Edwards, 2014). This is an emerging area of research over the next decade.

To date, the debate in universities as to whether to measure the student experience of learning as primarily the processes which support it, or the outcomes which are the result of it, has landed firmly on the latter. However, as the processes leading to quality outcomes become more and more complex because of the increasing number of variables involved, the case for measuring how the quality of processes contribute to the quality of outcomes is growing (Ellis & Goodyear, 2019). In a sense, while we might know what quality outcomes look like, if we do not understand how to get there, we will not know how to remedy a situation. Consequently, it is likely that we need to also understand and measure those processes which are most likely to result in quality outcomes, not just the outcomes alone.

### The pandemic of 2020–2022

At the time of writing this manuscript, the world is still in the grips of the COVID-19 pandemic which is likely to last at least another couple of years. The practical impact of the pandemic has motivated universities across the world to move their learning and teaching experience and course delivery online for health and logistical reasons. In many cases, this commenced within a rapid timeline in the first half of 2020 (Flores et al, 2022; Guppy et. al., 2022). Within the journal *Higher Education,* emerging research of the impact of the pandemic on the learning and teaching experience has considered the following: the impact on the learning and teaching experience from rising expectations of technology and learning off campus (Guppy et. al., 2022; Nordmann et. al., 2022); the accompanying pressure what can be considered appropriate conduct and etiquette for experiences of learning which are predominantly online (Oksanen, et. al., 2022); the academic legitimacy and possibilities of a mobile experience (Tzanakou & Henderson, 2021); the reduction in participation of international students in higher education (Netz, 2021 Whatley & Castiello-Gutiérrez, 2022); and the financial impact of the pandemic on the higher education sector (Komljenovic, 2020; Watermeyer, et. al., 2021).

One way of describing the outcomes of these changes is to say there has been a world-wide transformation of course delivery of university degrees requiring students and teachers, administrators, education leaders and whole institutions to adapt their approaches to education processes and outcomes to an online context. It is too early to say exactly what the outcomes of this will mean in the medium term for a typical university student experience of learning in a post-pandemic context; however, it is probably safe to say that there will be increased understanding from students, teachers and university leaders about what can be achieved and expected from blended experience of learning in terms of both efficiencies and improvements to learning.

## Conclusions

The purpose of this review is to give a taste of the depth and breadth of the contribution of *Higher Education* to international debates on learning and teaching over the last five decades. The themes chosen have been the *what* and *how* of learning and teaching innovation and how these key aspects of student learning are related to each other and to the learning outcomes of students in an international context moving out of a pandemic towards a new equilibrium for learning and teaching. While this synoptic structure is a strength for the coherence of the review, it is also a limitation of it. In any short manuscript, it is not possible to produce an exhaustive and completely comprehensive review of fifty years of research. Neither is it possible to reveal the widespread impact all this research has had on research in other journals and the student, teacher and educational leader experience more generally. Despite these limitations, this review *does* provide a useful basis upon which to discern key issues currently occupying international cutting-edge research into learning and teaching, how we arrived here and what themes and foci are likely to benefit students, teachers, university leaders and the universities in which they work in the next decade and more. Debate will continue about the most appropriate outcomes of a university education and how best to enable those outcomes through effective learning and teaching processes.

Given where we’ve come from, it is clear that innovation in learning and teaching is a continuously important phenomenon in the higher education sector and is driven by an increasing number of forces. The overall impact of this change is a growth in complexity of the student experience, in part contributed to by an integrated use of material elements such as technologies in the learning experience. Trying to understand the respective contribution of, and interdependencies amongst, students, teachers, course designs, technologies and other aspects of learning environments highlights the complexity of the current issues facing researchers. It is hard to isolate and identify which aspects of the learning experience should be attenuated if improvements to outcomes are sought. New methods of investigating and evaluating innovation in learning and teaching will continue to be necessary in the sector to reduce uncertainty and enhance learning and teaching, particularly if there are deterministic associations amongst the *what* and *how* of learning that lead to qualitatively better outcomes for students and society. The journal *Higher Education* will continue to play an important and pivotal role in helping to shape these debates in order to push excellence in the field of learning and teaching research onwards.
